# A dosimetric analysis of respiration-gated radiotherapy in patients with stage III lung cancer

**DOI:** 10.1186/1748-717X-1-8

**Published:** 2006-03-31

**Authors:** René WM Underberg, John R van Sörnsen de Koste, Frank J Lagerwaard, Andrew Vincent, Ben J Slotman, Suresh Senan

**Affiliations:** 1 Department of Radiation Oncology, VU University medical center, de Boelelaan 1117, 1007 MB, Amsterdam, The Netherlands; 2 Department of Bioinformatics, Antoni van Leeuwenhoek hospital, Plesmanlaan 121, 1066 CX, Amsterdam, The Netherlands

## Abstract

**Background:**

Respiration-gated radiotherapy can permit the irradiation of smaller target volumes. 4DCT scans performed for routine treatment were retrospectively analyzed to establish the benefits of gating in stage III non-small cell lung cancer (NSCLC).

**Materials and methods:**

Gross tumor volumes (GTVs) were contoured in all 10 respiratory phases of a 4DCT scan in 15 patients with stage III NSCLC. Treatment planning was performed using different planning target volumes (PTVs), namely: (i) PTV_routine_, derived from a single GTV plus 'conventional' margins; (ii) PTV_all phases _incorporating all 3D mobility captured by the 4DCT; (iii) PTV_gating_, incorporating residual 3D mobility in 3–4 phases at end-expiration. Mixed effect models were constructed in order to estimate the reductions in risk of lung toxicity for the different PTVs.

**Results:**

Individual GTVs ranged from 41.5 – 235.0 cm^3^. With patient-specific mobility data (PTV_all phases_), smaller PTVs were derived than when 'standard' conventional margins were used (p < 0.001). The average residual 3D tumor mobility within the gating window was 4.0 ± 3.5 mm, which was 5.5 mm less than non-gated tumor mobility (p < 0.001). The reductions in mean lung dose were 9.7% and 4.9%, respectively, for PTV_all phases _versus PTV_routine_, and PTV_gating _versus PTV_all phases_. The corresponding reductions in V_20 _were 9.8% and 7.0%, respectively. Dosimetric gains were smaller for primary tumors of the upper lobe versus other locations (p = 0.02). Respiratory gating also reduced the risks of radiation-induced esophagitis.

**Conclusion:**

Respiration-gated radiotherapy can reduce the risk of pulmonary toxicity but the benefits are particularly evident for tumors of the middle and lower lobes.

## Background

As lung tumors can show significant respiration-induced motion [1,2], sufficient margins have to be added to account for target mobility in radiotherapy planning [3]. For stage I non-small cell lung cancer (NSCLC), commonly used 'population-based' margins result in unnecessary irradiation of significant amounts of normal tissue [2,4], thereby increasing the risk of toxicity. For stage III NSCLC, local control after radiotherapy is poor [5,6], and both radiation dose-escalation and concurrent chemo-radiotherapy schemes have been used in an attempt to improve local control. However, such approaches can increase late radiation-induced toxicity [7-11], and reductions in treated volumes may help in reducing toxicity.

Respiration-correlated (or 4D) CT scans permit an individualized assessment of tumor mobility [12]. Respiratory gating enables smaller target volumes to be used as treatment delivery is restricted to predetermined phases of respiration, during which tumor mobility is relatively limited. However, planning and delivery of gated radiotherapy requires reliable data on intra- and inter-fractional tumor mobility. In stage I NSCLC, individualized planning target volumes (PTVs) that incorporated all tumor mobility or the residual mobility in three end-expiratory phases allowed for mean PTV reductions of 48.2% and 33.3%, respectively, relative to standard margins [2]. The magnitude of benefit that can be achieved with gating in stage I tumors was found to correlate with the extent of mobility.

A recent study in patients with stage III NSCLC reported that limited decreases in lung doses could be achieved with gating, but only if GTVs did not exceed 100–150 cm^3 ^[13]. However, tumor mobility in this study was derived from breath-hold CT scans at full inspiration and tidal end-expiration. A more realistic analysis requires knowledge of mobility in all phases of quiet respiration, i.e. 4DCT data. We retrospectively analyzed 4D datasets in order to determine the geometric and dosimetric benefits of respiration-gated radiotherapy in stage III NSCLC.

## Methods

The 4DCT scans of 15 consecutive patients with stage III NSCLC who were treated with conventionally fractionated involved-field radiotherapy at the VU University medical center, were retrospectively analyzed. The primary tumors were located in the upper (n = 9), middle (n = 2) and lower lobe (n = 4), respectively. Patient characteristics are summarized in Table 1. All patients had at least one metastatic N2-3 lymph node, and nodal locations were described according to Mountain et al. [14].

**Table 1:** Patient characteristics

Patient	TNM-stage	Primary tumor location	Lymph node locations	GTV (cc)
1	T_2_N_2_M_0_	Right upper lobe	2R, 4R	101.4
2	T_3_N_2_M_0_	Left upper lobe	4L	231.6
3	T_2_N_3_M_0_	Right upper lobe	4R, 4L	63.6
4	T_2_N_2_M_0_	Right lower lobe	7	77.9
5	T_3_N_3_M_0_	Right upper lobe	4R, 4L	174.7
6	T_2_N_2_M_0_	Right lower lobe	4R	99.1
7	T_3_N_2_M_0_	Right lower lobe	4R, 7	61.7
8	T_2_N_2_M_0_	Right upper lobe	4R	74.8
9	T_1_N_3_M_0_	Right middle lobe	4R, 4L	71.8
10	T_2_N_2_M_0_	Left lower lobe	4L, 7	180.9
11	T_4_N_3_M_0_	Right upper lobe	4L	154.6
12	T_2_N_2_M_0_	Left upper lobe	2L, 4L	41.5
13	T_2_N_3_M_0_	Right upper lobe	2R, 4R, 4L	84.2
14	T_3_N_2_M_0_	Left upper lobe	4L	235.0
15	T_3_N_2_M_0_	Right middle lobe	2R, 4R, 7	164.3

GTV = gross tumor volume

Lymph node locations are according to Mountain et al. [20].

### 4DCT scanning procedure

Patients were immobilized in the supine position, with the arms positioned above the head on an adjustable arm support and a knee rest device was used (Posirest-2 and Kneefix cushion device, Sinmed, Reeuwijk, The Netherlands). A 4DCT thoracic scan was performed during uncoached quiet respiration on a 16 slice CT scanner. A respiratory monitoring system (Respiratory Position Management, Varian Medical Systems, Palo Alto, CA) was used for recording the breathing pattern. A lightweight block containing two reflective markers is placed on the upper abdomen, typically halfway between the xiphoid and umbilicus, and infrared light from an illuminator is reflected from the markers, and captured by a camera.

Our 4DCT protocol for scanning lung tumors has been reported in detail previously [12]. Briefly, 8 contiguous slices of 2.5 mm are generated for a 2 cm total longitudinal coverage per gantry rotation with the scanner operated in axial cine mode. Other scanning parameters include 140 KV, 95 mA and tube rotation set closest to 1/10 of the average breathing cycle time to allow high temporal and spatial resolution. With the scanner couch in static mode, data is acquired for at least the duration of one full respiratory cycle, after which the couch advances to the next position. Data acquisition ceases during the couch movement, and a full 4DCT scanning procedure of the thorax generally takes about 90 seconds. The radiation exposure from the 4DCT acquisition is about 52.7 mGy in CTDI_vol _when the patients' breathing cycle is 4 sec, which is about 6 times the dose of our single conventional helical CT scan procedure. Retrospective sorting of the images into spatio-temporally coherent volumes is performed using 4D software (Advantage 4D, GE Medical Systems, Waukesha, WI). Each reconstructed image is assigned to a specific respiratory phase (or 'bin') based on the temporal correlation between surface motion and data acquisition, and 10 respiratory phases are generated. The actual clinical treatment of these patients was based upon non-gated treatment delivery to a PTV that incorporated mobility seen in all phases of respiration (PTV_all phases_).

In this retrospective analysis, the bins representing all 10 phases of respiration were imported into the radiotherapy planning system (Eclipse version 6.5, Varian Medical Systems, Palo Alto, CA). All ten 3D data sets derived from a 4DCT scan shared DICOM coordinates, which simplified image registration of the data sets using the "shared DICOM coordinates" option. After image registration, the position of a vertebra at the level of the primary tumor was checked to ensure that no patient movement had occurred during the 4DCT acquisition procedure.

### Defining the 'gating- window'

As gating will prolong treatment delivery, it is common to use a 'gate' that allows a duty cycle of at least between 20–40% of respiration [15]. In order to identify the gate, all ten 4DCT bins were imported into the '4D Review' application in the Advantage workstation where 3 or 4 consecutive 'gating bins' in expiration were simultaneously viewed in axial, frontal and sagittal reconstructions. A 'gating window' was identified at end-expiration in which mobility of the primary tumor and/or hilus was minimal. The use of longer gating windows improve the efficiency of treatment delivery, and a total of 4 phases were selected in 4 patients as review of the 4DCT movies suggested limited mobility of the primary tumor within in these 'gates'.

### Deriving target volumes and contouring critical structures

Gross tumor volumes (GTVs) were contoured in each of the 10 phases of a 4DCT using standardized lung and mediastinal window level settings by one clinician in order to minimize contouring variations. The use of involved-field radiotherapy is standard practice at our center for the treatment of stage III NSCLC [16], and the GTV included the primary tumor, ipsilateral hilus, lymph nodes with a short axis diameter of 1.0 cm or greater and/or FDG-PET positive lymph nodes. All contours were automatically projected onto the second 'bin' in the middle of the gating window. Similarly, the spinal cord and esophagus were also contoured in this specific 'gating bin'. In six patients, the primary tumor and the hilus were contoured as a single entity as the two structures were adjacent to each other.

For all 15 patients, three PTVs were generated:

(i) PTV_routine_, derived from a phase at the center of the 'gating window', i.e from a single component CT scan. An isotropic margin of 1.5 cm (but 2.0 cm for cranial and caudal margins in lower lobe tumors) was added to the GTV in order to account for microscopic extension, mobility and set-up inaccuracies;

(ii) PTV_all phases_, which consisted of the volume encompassing all ten GTVs (i.e. all respiration-induced mobility), plus an isotropic margin of 1.0 cm for microscopic extension and set-up inaccuracies;

(iii) PTV_gating_, consisting of the volume encompassing 3–4 GTVs in the gating window, plus an isotropic margin of 1.0 cm for microscopic extension and set-up inaccuracies.

### Analysis of target mobility

A 'bounding box' technique was used to assess the mobility of GTVs, as was previously reported [17]. Briefly, the approach involves fitting a rectangular box to each target volume, and changes in the position of a moving target in the X-, Y- and Z-axes are derived from the corresponding sides of the box. The overall 3D displacement of each GTV was derived using Pythagoras's algorithm.

The following analyses were performed: (i) mobility of individual GTVs, derived from displacement of a single GTV (GTV_single phase_) within GTV _all phases_, (ii) the reduction in mobility achieved with the use respiratory gating, derived from calculating the displacement of the GTV_gating _within GTV_all phases_, and (iii) residual GTV mobility within gating windows, derived from displacement of the GTV_single phase _within GTV_gating_.

### Treatment planning and dosimetric analysis

Treatment planning was performed on each PTV_routine_, PTV_all phases_and PTV_gating_, all of which were projected onto a single end-expiratory CT set. The study patients were treated using two different fractionation schemes, namely 60 Gy in 30 fractions and 46 Gy in 23 fractions. The latter was a dose used for pre-operative concurrent chemo-radiotherapy. For both schemes, the PTVs received at least 95% of the prescribed dose, and a dose maximum of 107% of the prescribed dose was accepted unless a higher dose maximum was located within the PTV. The V_20 _values had to be less than 35%, and dose to spinal cord less 50 Gy. An initial treatment plan was derived for PTV_routine_, and treatment plans for PTV_all phases _and PTV_gating _were then derived by shrinking the fields of the initial plan, without other changes to the beam setup.

Dose-volume histograms (DVH) were computed for the total lung minus the volume of PTV_gating _in order to generate a reference lung volume that was copied to each treatment plan. The maximum spinal cord dose and predictors of lung toxicity, the V_20 _and mean lung dose (MLD) [18], were evaluated for each plan. The length of the esophageal circumference encompassed by the 40 Gy and 50 Gy isodose was obtained by visually scrolling through axial CT slices.

### Statistical analysis

The differences between the three PTVs and the reduction in GTV mobility achieved with gating were evaluated using the Hodges-Lehman non-parametric test. The existence of a correlation between the reduction in GTV mobility with respiratory gating and the dosimetric gains achieved, were evaluated using the Spearman's non-parametric correlation test. In addition, the dosimetric data was analyzed for differences according to tumor stage and tumor location (upper vs. non-upper lobe. All statistical analyses were conducted using the Cytel Studio's StatXact-6 software (Version 6.2.0).

Finally, mixed effect models were constructed (SAS version 8.02 and S-plus version 6.2) in order to estimate the general dosimetric gains that can be achieved (PTV_routine _vs. PTV_all phases_; PTV_routine _vs. PTV_gating_; PTV_all phases _vs. PTV_gating_) on the V_20 _and MLD, as well as on critical structures (esophagus and spinal cord).

## Results

### Volumetric comparison of PTVs

**Figure 1:**
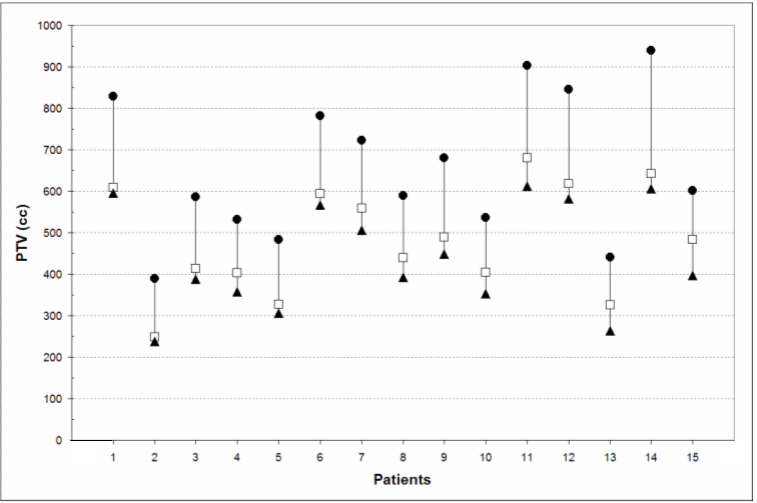
Absolute volumes of PTV_routine_(●), PTV_all phases_(□) and PTV_gating_(▲).

The individual GTVs ranged from 41.52 cc to 235.04 cc, and six GTVs were larger than 150 cc (Table 1). The PTV_routine _was the largest volume for all patients (Figure 1). Use of individualized margins led to significantly smaller volumes (PTV_all phases_) than with the use of standard margins (p < 0.001). The same was observed for PTV_gating _versus PTV_all phases_(p < 0.001).

### Mobility of target volumes

The mean mobility of GTV_single phase _within GTV_all phases _was 9.4 ± 6.3 mm (1 SD) and the data are summarized in Table 2. With a gating 'duty cycle' extending to 3–4 phases of the respiratory cycle, mean GTV mobility was reduced by 5.5 ± 3.8 mm (p < 0.001). The mean "residual" GTV mobility within the chosen gating window was 4.0 ± 3.5 mm (Table 2), and a residual GTV mobility that exceeded 5.0 mm was observed in 5 patients (33%), including 2 patients with upper lobe lesions. When only tumors of the middle and lower lobe were evaluated, the mean GTV mobility in all phases was 13.7 ± 7.1 mm, which decreased to 5.6 ± 4.6 mm with gating.

**Table 2:** GTVs and GTV mobility.

Patient	GTV_all phases _(cc)	GTV_gating _(cc)	Ungated GTV mobility (mm)	Residual GTV mobility (mm)
1	126.9	104.1	5.6	2.2
2	247.4	239.0	1.0	0.0#
3	76.3	68.1	11.2	5.8
4	132.3	90.6	17.3	6.4
5	201.0	183.4	8.2	3.2#
6	128.6	112.5	22.6	14.0#*
7	98.4	66.8	18.2	5.9
8	112.7	81.9	10.9	2.0
9	109.8	79.8	8.4	1.4
10	216.3	193.2	3.0	2.0
11	199.8	162.5	8.7	4.6
12	49.7	44.0	1.0	1.0#
13	97.7	89.0	8.4	6.4
14	254.0	239.3	4.6	1.0
15	240.7	193.3	12.6	3.6
				
Mean	152.8	129.8	9.4	4.0
SD	67.1	65.5	6.3	3.5

# gating-window incorporated four successive phase bins.

* mainly due to residual nodal mobility in the mediastinum

### Treatment planning

Treatment was planned to a dose of 60 Gy for 11 of the 15 patients. The other four had lesions of the upper lobe that extended to the contralateral mediastinum, and all these patients underwent pre-operative chemo-radiotherapy to 46 Gy. Reduction in the risk of pulmonary toxicity with gating was assessed using dedicated statistical models for all patients (n = 15) on mean lung dose (MLD) and in 14 patients with respect to the volume of lungs receiving at least 20 Gy (V_20_). One patient was excluded from the V_20 _model as the V_20 _values of the plans did not meet the V_20 _inclusion criteria, i.e. V_20 _(routine) > V_20 _(all phases) > V_20 _(gating). In this patient with a right lower lobe tumor, the V_20 _(all phases) was larger than the V_20 _(routine), which was the result of substantial lateral mobility of the mediastinal lymph nodes. Nevertheless, the PTV_all phases _plan did meet the inclusion criteria used in the MLD model, i.e. MLD (routine) > MLD (all phases) > MLD (gating).

The mixed effect model showed that the MLD was reduced by 9.7%, 4.9% and 14.2%, respectively, for PTV_all phases _versus PTV_routine_, PTV_gating _versus PTV_all phases _and PTV_gating _versus PTV_routine_. The corresponding figures for the V_20 _were 9.8%, 7.0% and 16.2% (Tables 3, 4). Upper lobe tumors showed a significantly lower dosimetric gain than tumors located in other lobes (p = 0.02). When the analysis was restricted to lower and middle lobe tumors, the corresponding figures for V_20 _reduction were 7.2%, 10.1% and 16.5%, and for reduction in MLD 9.9%, 6.4% and 15.7%, respectively. Tumor stage had no significant influence on the dosimetric improvements achieved with respiratory gating.

**Table 3:** Reduction in mean lung dose (Gy) using different PTV definitions.

	Mean lung dose (Gy)	Absolute (Gy) and relative (%) reduction in mean lung dose		
Patient	PTV_routine_	PTV_all phases _vs. PTV_routine_	PTV_gating _vs. PTV_routine_	PTV_gating _vs. PTV_all phases_

1	25.2	2.7 (10.7%)	3.6 (14.3%)	0.9 (4.0%)
2	20.6	1.6 (7.8%)	2.4 (11.7%)	0.8 (4.2%)
3	18.9	1.6 (8.5%)	2.2 (11.6%)	0.6 (3.5%)
4	23.0	1.3 (5.7%)	3.0 (13.0%)	1.7 (7.8%)
5	12.8	1.1 (8.6%)	1.5 (11.7%)	0.3 (2.6%)
6	18.7	1.7 (9.1%)	2.5 (13.4%)	0.8 (4.7%)
7	23.1	3.1 (13.4%)	4.2 (18.2%)	1.1 (5.5%)
8	10.4	0.7 (6.7%)	0.7 (6.7%)	0.0 (0.0%)
9	19.3	1.1 (5.7%)	2.7 (14.0%)	1.6 (8.8%)
10	20.2	3.0 (14.9%)	3.8 (18.8%)	0.8 (4.7%)
11	17.2	1.0 (5.8%)	2.1 (12.2%)	1.1 (6.8%)
12	19.5	3.2 (16.4%)	3.3 (16.9%)	0.1 (0.6%)
13	14.2	1.2 (8.5%)	2.4 (16.9%)	1.2 (9.3%)
14	15.7	2.1 (13.4%)	2.6 (16.6%)	0.5 (3.7%)
15	27.8	3.0 (10.8%)	4.7 (16.9%)	1.7 (6.9%)
				
Mean	19.1	1.9 (9.7%)	2.8 (14.2%)	0.9 (4.9%)
SD	4.6	0.9 (3.4%)	1.0 (3.2%)	0.5 (2.7%)

PTV = planning target volume

**Table 4:** Reduction in V_20 _(%) using different PTV definitions.

	V20 (%)	Absolute V20 (%) and relative (%) reduction in V20		
Patient	PTV_routine_	PTV_all phases _vs. PTV_routine_	PTV_gating _vs. PTV_routine_	PTV_gating _vs. PTV_all phases_

1	38.0	4.1 (10.8%)	5.1 (13.4%)	1.0 (2.9%)
2	27.1	2.2 (8.1%)	3.5 (12.9%)	1.3 (5.2%)
3	29.0	2.0 (6.9%)	2.8 (9.7%)	0.8 (3.0%)
4	31.0	3.8 (12.3%)	6.7 (21.6%)	2.9 (10.7%)
5	26.1	2.2 (8.4%)	2.9 (11.1%)	0.7 (2.9%)
6	28.9	-1.5 (-5.2%)	0.1 (0.3%)	1.6 (5.3%)
7	26.2	0.9 (3.4%)	4.3 (16.4%)	3.4 (13.4%)
8	20.5	3.7 (18%)	4.4 (21.5%)	0.7 (4.2%)
9	28.2	1.7 (6.0%)	4.8 (17.0%)	3.1 (11.7%)
10	27.5	4.2 (15.3%)	6.5 (23.6%)	2.3 (9.9%)
11	27.6	2.4 (8.7%)	4.9 (17.8%)	2.5 (9.9%)
12	25.5	4.4 (17.3%)	4.9 (19.2%)	0.5 (2.4%)
13	28.8	2.9 (10.1%)	6.0 (20.8%)	3.1 (12.0%)
14	23.9	3.7 (15.5%)	4.2 (17.6%)	0.5 (2.5%)
15	42.8	5.0 (11.7%)	8.6 (20.1%)	3.6 (9.5%)
				
Mean	28.7	2.8 (9.8%)	4.6 (16.2%)	1.9 (7.0%)
SD	5.4	1.7 (5.9%)	2.0 (6.0%)	1.2 (4.1%)

**Figure 2:**
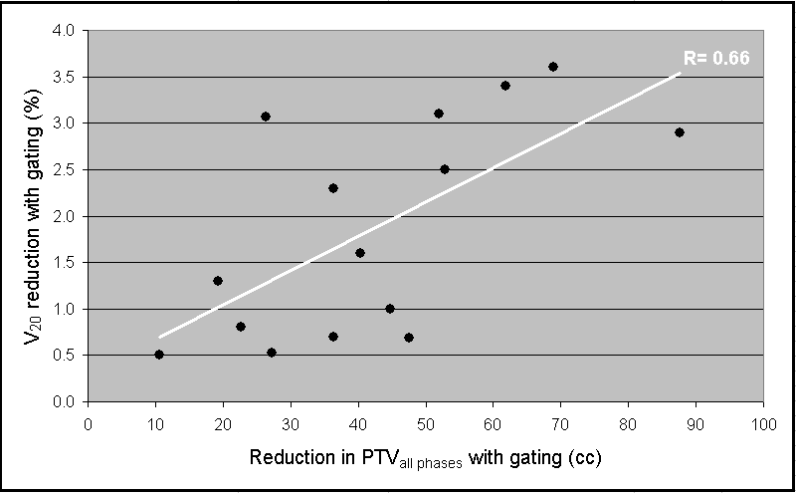
a-b Dosimetric correlation between (a) PTV reduction and V20 reduction, and (b) PTV reduction and MLD reduction.

**Figure 3:**
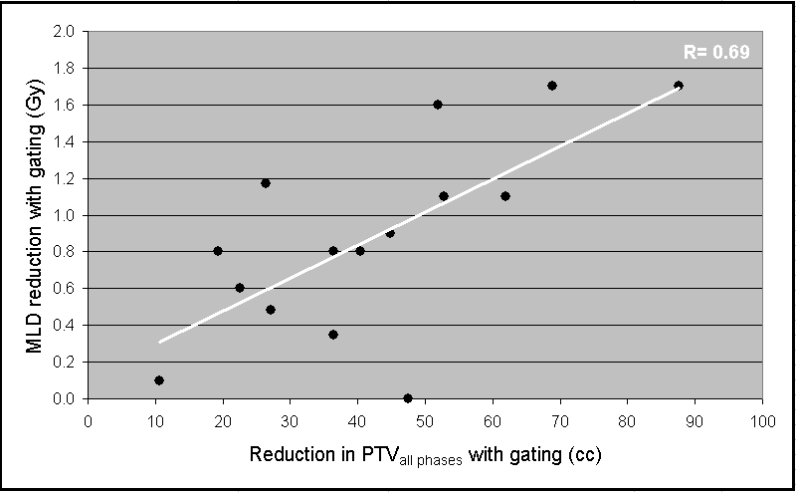
a-b Dosimetric correlation between (a) PTV reduction and V20 reduction, and (b) PTV reduction and MLD reduction.

As could be expected, a strong correlation was observed between the reduction in PTV observed with respiratory gating and the reduction in GTV mobility (Spearman's Non-parametric correlation test ρ = 0.78, linear regression coefficient = 4.3 (p < 0.001)). In addition, a correlation could be demonstrated between the reduction in PTV with gating and reductions in both in V_20 _and MLD values (Figure 2 and 3).

### Esophageal and spinal dose parameters

With the use of involved-field radiotherapy, only 11 patients received an esophageal circumference dose of at least 40 Gy. The mean circumferential length receiving a dose of 40 Gy was 6.8 ± 2.3 cm for PTV_routine_. Use of PTV_all phases _reduced this value by a mean of 0.8 cm, and the reduction with PTV_gating _was 1.2 cm, both proved significant (Satterthwaite approximation for the denominator degrees of freedom used to give accurate F approximations). A circumferential dose of 50 Gy was seen in 8 patients, and the mean esophageal circumference receiving 50 Gy was 5.3 ± 2.0 cm for PTV_routine_. Use of PTV_all phases _and PTV_gating _reduced this value by a mean of 1.8 and 2.3 cm, respectively (p < 0.001, Page T: k-tuple non-parametric exact test for grouped data).

The maximum spinal cord dose was 44.7 ± 7.4 Gy for involved-field treatment plans generated for PTV_routine_, and non-significant reductions to 43.7 ± 7.7 Gy and 42.8 ± 8.8 Gy were seen for plans based on PTV_all phases _and PTV_gating_.

## Discussion

Attempts to improve local control in stage III NSCLC have been confounded by an increased incidence of treatment-related toxicity, including unexpected late toxicities such as symptomatic bronchial stenosis, mediastinal fibrosis and stenosis of the pulmonary artery [9,11]. The reporting of significant lung tumor motion has led to large margins being added to the clinical target volume to ensure adequate dose coverage. As such, respiration-gated radiotherapy is an approach that can reduce the volume of irradiated normal tissue.

This study in 15 consecutive patients with stage III NSCLC highlights the volumetric and dosimetric gains that can be achieved using individualized PTVs that incorporate all tumor motion and also respiration-gated PTVs in expiratory phases. Of note is the finding that significant gains are attained simply by using individualized 4D mobility margins (i.e. PTV_all phases_) instead of PTVs based upon standard margins of 1.5 cm. The additional reductions in lung toxicity parameters achieved with respiratory gating were generally modest, but strongly correlated with the reduction in tumor mobility achieved with gating. Careful patient selection appears required to establish the optimal benefit.

Just as for stage I NSCLC [2], the larger PTV_routine _did not achieve coverage of all mobility in 7 of the 15 patients with tumors in upper- (n = 2), middle- (n = 2), and lower lobes (n = 3). For the upper lobe tumors, the mobility of mediastinal lymph nodes appeared to be inadequately incorporated using standard margins; for the middle- and lower lobe tumors this was the case for either the subcarinal nodes (n = 2), mediastinal lymph nodes (n = 3) or the primary tumor (n = 2).

This analysis of 4DCT data revealed that reductions in both V_20 _and MLD can be achieved even when GTVs exceed 150 cc, but that this was restricted to tumors located in middle and lower lobe. For the latter, the mean reduction in V_20 _was 10.1 ± 2.7% for PTV_gating _relative to PTV_all phases_. In contrast, recent reports suggested that dosimetric benefits for gated radiotherapy were restricted to patients whose GTVs were 150 cc or less, and which also exhibited significant mobility [13]. The mean mobility in our 15 patients (9.4 ± 6.3 mm) was similar to that of the 20 patients in the abovementioned report (9.4 ± 4.1 mm).

The potential benefits of respiratory gating must be viewed in the light of the residual uncertainties during gated delivery, for example the correlation between the PTV and external respiratory surrogates [19]. As changes in target volumes for lung cancer have been observed during both stereotactic radiotherapy [20] and conventionally-fractionated radiotherapy [21], the relationship between external markers and the PTV may have to be re-established during treatment. Ongoing developments in volumetric imaging at the treatment unit may increase the accuracy of respiration-gated radiotherapy.

Another approach explored for reducing toxicity is intensity-modulated radiotherapy (IMRT). A planning study comparing IMRT and 3D conformal radiotherapy in 41 patients with locally-advanced stage NSCLC reported a median reduction in V_20 _of 10% and a reduction in MLD of ≥2 Gy [22]. However, 4D treatment planning for conformal radiotherapy and IMRT is in its infancy [23].

A limitation of our study is the fact that the esophagus was contoured in only one phase of respiration position. Recent data suggests that respiration-induced mobility of the lower esophagus can occur [24], which can confound our conclusions about the dosimetric impact of gated delivery.

## Conclusion

Our analysis of 4DCT data indicates that individualized 4DCT-based PTVs ensure optimal target coverage with minimal irradiation of normal tissues in patients with stage III NSCLC. Respiration-gated radiotherapy can further reduce the risk of pulmonary toxicity, particularly for tumors located in the middle and lower lobes.

## Competing interests

1. The VU University medical center has research collaborations with Varian Medical Systems (Palo Alto, CA) and GE Healthcare (Waukesha, WI) in the field of 4DCT scanning and respiration-gated radiotherapy.

2. S. Senan and F.J. Lagerwaard have received speaker's fees from GE Healthcare.

## Authors' contributions

S.S. and J.VSDK designed the study, analysed all study data and prepared the final version of the manuscript. R.U. generated patient contours, performed treatment planning and initial analysis, and he generated the first drafts of the manuscript. F.L. analysed all study data and prepared the final manuscript. B.S. was involved in study design and drafting of the manuscript. A.V. performed statistical analysis of the study data and drafting of the manuscript.
